# Polyphenol Levels Are Inversely Correlated with Body Weight and Obesity in an Elderly Population after 5 Years of Follow Up (The Randomised PREDIMED Study)

**DOI:** 10.3390/nu9050452

**Published:** 2017-05-03

**Authors:** Xiaohui Guo, Anna Tresserra-Rimbau, Ramón Estruch, Miguel A. Martínez-González, Alexander Medina-Remón, Montserrat Fitó, Dolores Corella, Jordi Salas-Salvadó, Maria Puy Portillo, Juan J. Moreno, Xavier Pi-Sunyer, Rosa M. Lamuela-Raventós

**Affiliations:** 1 Department of Nutrition, Food Science and Gastronomy, XaRTA, INSA-UB, School of Pharmacy and Food Science, University of Barcelona, 08028 Barcelona, Spain; guoxiaohui1130@gmail.com (X.G.); annatresserra@ub.edu (A.T.-R.); jjmoreno@ub.edu (J.J.M.); 2 CIBEROBN Fisiopatología de la Obesidad y Nutrición, Instituto de Salud Carlos III, 28029 Madrid, Spain; restruch@clinic.ub.es (R.E.); mamartinez@unav.es (M.A.M.-G.); amedina@ub.edu (A.M.-R.); mfito@imim.es (M.F.); dolores.corella@uv.es (D.C.); jordi.salas@urv.cat (J.S.-S.); mariapuy.portillo@ehu.es (M.P.P.); 3 Department of Internal Medicine, Hospital Clínic, IDIBAPS, University of Barcelona, 08036 Barcelona, Spain; 4 Department of Preventive Medicine and Public Health, School of Medicine & IdiSNA (Institute for Health Research), University of Navarra, 31080 Pamplona, Spain; 5 Department of Nutrition, Harvard TH Chan School of Public Health, Boston, MA 02115, USA; 6 Cardiovascular Risk and Nutrition Research Group (CARIN, Regicor Study Group), IMIM (Hospital del Mar Medical Research Institute), 08003 Barcelona, Spain; 7 Department of Epidemiology, Preventive Medicine and Public Health, School of Medicine, University of Valencia, 46010 Valencia, Spain; 8 Human Nutrition Unit, University Hospital of Sant Joan de Reus, Department of Biochemistry and Biotechnology, Faculty of Medicine and Health Sciences, IISPV, Rovira i Virgili University, 43201 Reus, Spain; 9 Nutrition and Obesity Group, Department of Nutrition and Food Science, Faculty of Pharmacy and Lucio Lascaray Research Institute, University of País Vasco (UPV/EHU), 01006 Vitoria, Spain; 10 New York Obesity Research Center, Department of Medicine and Institute of Human Nutrition, Columbia University, New York, NY 10032, USA; fxp1@columbia.edu

**Keywords:** overweight, obesity, polyphenol, urine, PREDIMED

## Abstract

Overweight and obesity have been steadily increasing in recent years and currently represent a serious threat to public health. Few human studies have investigated the relationship between polyphenol intake and body weight. Our aim was to assess the relationship between urinary polyphenol levels and body weight. A cross-sectional study was performed with 573 participants from the PREDIMED (Prevención con Dieta Mediterránea) trial (ISRCTN35739639). Total polyphenol levels were measured by a reliable biomarker, total urinary polyphenol excretion (TPE), determined by the Folin-Ciocalteu method in urine samples. Participants were categorized into five groups according to their TPE at the fifth year. Multiple linear regression models were used to assess the relationships between TPE and obesity parameters; body weight (BW), body mass index (BMI), waist circumference (WC), and waist-to-height ratio (WHtR). After a five years follow up, significant inverse correlations were observed between TPE at the 5th year and BW (β = −1.004; 95% CI: −1.634 to −0.375, *p* = 0.002), BMI (β = −0.320; 95% CI: −0.541 to −0.098, *p* = 0.005), WC (β = −0.742; 95% CI: −1.326 to −0.158, *p* = 0.013), and WHtR (β = −0.408; 95% CI: −0.788 to −0.028, *p* = 0.036) after adjustments for potential confounders. To conclude, a greater polyphenol intake may thus contribute to reducing body weight in elderly people at high cardiovascular risk.

## 1. Introduction

Overweight and obesity have been steadily increasing in recent years and currently represent a serious threat to public health [1]. In 2014, more than 1.9 billion adults were overweight worldwide, and of these over 600 million were obese [2]. With nearly three million adults dying each year as a result of being overweight or obese, the impact of obesity on morbidity, mortality, and health care costs is very high [3]. Lifestyle and dietary habits are key determinants in the prevalence of obesity [4,5,6].

Polyphenols, the most abundant antioxidants in nature, are widely distributed in plant-derived foods such as vegetables, fruits, seeds, coffee, wine, and tea [7]. Only a few human studies have reported a relationship between polyphenol intake and body weight, even though obesity is considered a major independent risk factor for various chronic diseases [8,9]. Evidence for the effects of polyphenols on obesity parameters in humans is inconsistent, possibly due to divergence among study designs, characteristics of the participants, and metabolic pathways. Although some intervention clinical trials with polyphenol-enriched food or polyphenol extracts do not show any effect on weight or waist circumference [10,11,12], other studies have reported that polyphenols reduce body weight and increase energy expenditure [13,14,15,16]. The oral bioavailability of polyphenols is particularly important because, after being modified and metabolized by enzymes, their concentration in tissues and biological fluids is quite low [9,12,15,17]. There is therefore a need for a biomarker to accurately reflect polyphenol concentration after their absorption and metabolism. 

Polyphenol plasma levels or total urinary polyphenol excretion, considered in recent years as a reliable biomarker of total polyphenol intake, has been correlated with dietary polyphenol intake, and has been applied to explore associations between polyphenol intake and several chronic disease risk parameters [18,19,20,21]. Thus, the objective of the current study was to assess the associations between total polyphenol intake, measured by total urinary polyphenol excretion (TPE), and obesity parameters in an elderly population at high cardiovascular risk after five years of follow up.

## 2. Materials and Methods

The protocol for this trial and supporting Strengthening the Reporting of Observational studies in Epidemiology (STROBE) checklist are available as supporting information.

### 2.1. Ethics Statement

All participants provided informed consent. The Institutional Review Board (IRB) of the Hospital Clinic (Barcelona, Spain), accredited by the US Department of Health and Human Services (DHHS) update for Federal wide Assurance for the Protection of Human Subjects for International (Non-US) Institutions #00000738, approved the study protocol on 16 July 2002. The authors confirm that all ongoing and related trials for this drug/intervention are carried out following the rules of the Declaration of Helsinki of 1975 and registered (ISRCTN35739639).

### 2.2. Subjects

Participants were drawn from the PREDIMED Study (‘Prevención con Dieta Mediterránea’ (Prevention with the Mediterranean Diet), ISRCTN35739639). The information in the registry was delayed after recruitment began, to be sure about the feasibility of the study protocol; we started the trial as a ‘pilot study’ on October 2003, and, once we were sure that the intervention protocol worked, we decided to submit the study protocol for registration (date of application: 2 September 2005). The protocol and recruitment method are reported in detail elsewhere [22].

The present study looks at 573 participants that were recruited in two centers, the Clinic Hospital of Barcelona and the University of Valencia, both in Spain, and all were followed-up after more than five years. The period of recruitment was from 2003 to 2006, and the average follow up was 5.9 years.

### 2.3. Nutritional Measurements

The selected participants were asked to complete some questionnaires: a validated 137-item food frequency questionnaire (FFQ) to assess dietary habits [23]; a 47-item general questionnaire aimed to summarize information about lifestyle, health condition, education, history of illnesses, and medication use; a 14 point questionnaire evaluating the degree of adherence to the Mediterranean diet [24]; and a validated Spanish version of the Minnesota Leisure-Time Physical Activity Questionnaire to record physical activity [25]. Nutrient intake was adjusted by calories using the residual method [26]. All questionnaires were administered and repeated yearly during the follow up by trained staff in face-to-face interviews.

### 2.4. Urine Samples

Spot urine samples from the participants were collected and coded at the clinic by a technician and then immediately shipped to a central laboratory to be stored at −80 °C until analyzed.

### 2.5. TPE Measurements 

The Folin-Ciocalteu method was applied to determine the content of TPE, using a clean-up procedure with solid phase extraction (SPE) performed in 96-well plate cartridges (Oasis MAX), which helped to remove urinary interferences. Finally, TPE was expressed as milligrams of gallic acid equivalent (GAE)/g of creatinine. All details have been previously described by Medina-Remón et al. [19].

### 2.6. Measurements

Weight and height were measured with calibrated scales and a wall-mounted stadiometer, respectively. Body mass index (BMI) was calculated as weight in kilograms divided by the square of height in meters. Waist circumference (WC) was measured midway between the lowest rib and the iliac crest. Waist-to-height ratio (WHtR) was calculated as the waist in centimeters divided by the height in meters. Blood pressure was determined in triplicate using a validated semi-automatic sphygmomanometer (Omron HEM-705CP, Tokyo, Japan) by trained nurses. Measurements were taken at three time points, separated by 2 min, while the participant was in a seated position after 5 min of rest [27]. Obesity is defined as BMI more than 30 kg/m^2^.

### 2.7. Statistical Analysis

Results were expressed as mean ± SD for continuous variables or percentages for categorical variables. Kolmogorov and Levene tests were applied to examine the normality distribution and skewness. All participants, including total subjects, males, and females were divided into five categories according to their TPE at the fifth year of follow up. Changes in nutrient intakes and key food consumption according to the FFQs were assessed with yearly repeated-measures analysis during the follow up period. A Bonferroni post-hoc test and paired *t*-test were used to compare each variable within and between groups.

Multiple linear regression models were used to assess the relationship between anthropometric parameters (Body weight (BW), BMI, WC, and WHtR) and quintiles of TPE at the fifth year, adjusted for potential confounders, including sex, age, intervention groups, smoking status (never, current, former), family history of coronary heart disease (CHD), physical activity, hypertension, diabetes, dyslipidemia, marital status (single, married, widowed), education level (primary school, high school, university), medication used (antihypertensive drugs, vitamins, insulin, oral hypoglycemic drugs, aspirin, or other antiplatelet drug supplements taken in the last month), recruitment centers, 14 unit Mediterranean diet score, and energy intake at baseline. Multiple logistic regression analyses were used to calculate the odds ratio (OR) for quintiles of TPE and obesity (BMI > 30 kg/m^2^). Models were adjusted for potential confounders as in linear regression analyses.

All analyses were performed using SPSS software V21.0 (SPSS Inc., Chicago, IL, USA,). All models were tested for the detection of outliers, multicollinearity, homoscedasticity, and normality and independence of errors. All statistical tests were two-tailed, and the significance level was *p* < 0.05. The detailed information of the participants is available as supporting information.

## 3. Results

**Figure 1:**
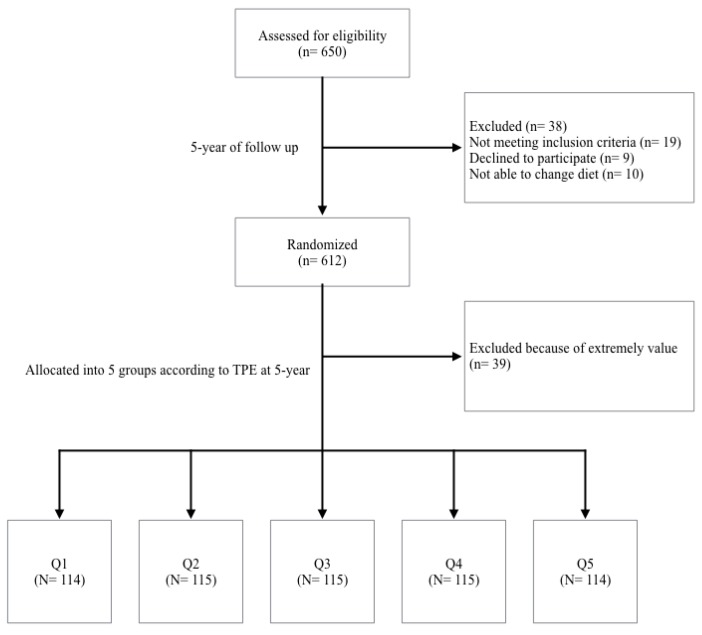
Flowchart of study participants.

A total of 650 subjects were randomly selected from two centers, the Hospital Clinic of Barcelona and the University of Valencia. From them, 38 were excluded because they did not meet the inclusion criteria during the intervention, and, after five years, 39 were excluded because their TPE concentrations were considered outliers, which was defined as any data point more than 1.5 interquartile ranges below the first quartile or above the third quartile; hence a total of 573 participants were finally included (Figure 1).

The baseline characteristics of participants grouped by quintiles of TPE at baseline are shown in Table 1. There were a total of 277 men and 296 women with a mean age of 66.2 ± 6.1 years and 68.3 ± 5.4 years, respectively. Of those participants, 41.5% had diabetes, 80.5% had hypertension, 66.8% had dyslipidemia, 16.9% were current smokers, and 37.5% had a family history of CHD. Compared with participants with the lowest TPE, those with higher TPE were more likely to be women, older, less likely to smoke, and also had lower body weight. Q4 shows the lowest prevalence of hypertension.

**Figure 2:**
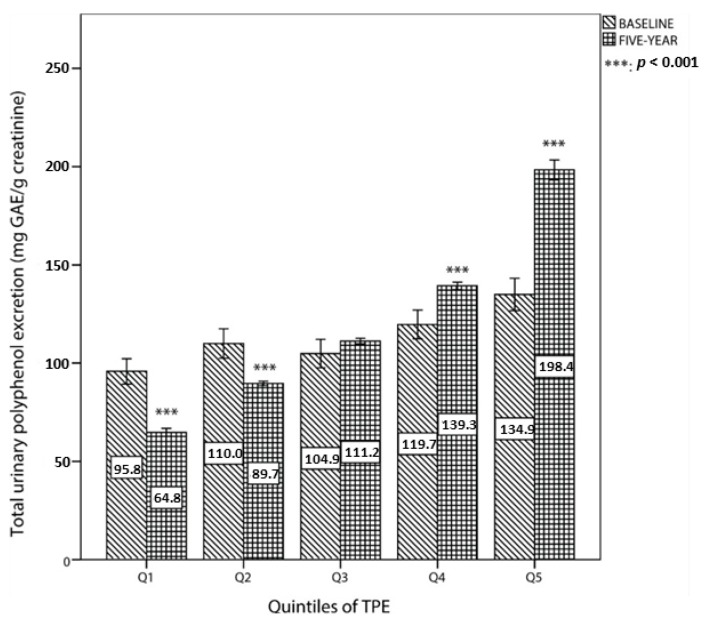
Total polyphenol excretion at baseline and at the fifth year of follow up by quintiles of TPE.

The comparison of total urinary polyphenol excretion between baseline and the fifth year of follow up by quintiles of TPE at the fifth year is shown in Figure 2. For the first two quintiles, TPE at baseline was significantly higher than at the fifth year. By contrast, TPE at the top two categories was higher than at baseline.

Table 2 summarizes information on key changes in food consumption during the intervention according to quintiles of TPE. As shown, at the end of the intervention, the consumption of most of the items belonging to a Mediterranean dietary pattern had increased significantly, including vegetables, fruits, fish, extra virgin olive oil, olive oil, nuts, coffee, and milk. However, the intake of wine decreased significantly, as well as intakes of cereals, meat, and pastries. Table 2 also shows changes in nutrient intake and degrees of adherence to a Mediterranean diet. Significant increments were observed in the consumption of total fat, fiber, polyunsaturated fatty acids (PUFA), monounsaturated fatty acids (MUFA), folic acid, potassium (K), and magnesium (Mg), while total carbohydrates, protein, cholesterol, sodium (Na), and saturated fatty acids (SFA) remained similar throughout.

The associations between TPE and obesity indexes were analyzed by linear regression models (Table 3). For total participants, significant inverse associations were found between quintiles of TPE at the fifth year and BW (β = −1.004; *p* = 0.002), BMI (β = −0.320; *p* = 0.005), WC (β = −0.742; *p* = 0.013), and WHtR (β = −0.408; *p* = 0.036) after adjustment for potential confounders. For males, inverse associations were found in BW (β = −0.959; *p* = 0.039) and BMI (β = −0.301; *p* = 0.034), while, for females, only an inverse association was found in BMI (β = −0.332; *p* = 0.046) after a full adjustment.

Table 4 shows the odds ratio (OR) and a 95% CI for obesity according to the quintile of TPE at the fifth year. In fully adjusted models, for total participants in the category of highest TPE had a lower prevalence of obesity (OR = 0.346, 95% CI = 0.176 to 0.178; *p*-trend = 0.039) than those in the lowest category. For males, compared with the reference group, the top quintile group (Q5) showed significant reduction in the prevalence of obesity (OR = 0.340, 95% CI = 0.146 to 0.792 in Model 2).

Table 5 shows the incidence of obesity after five years of intervention, conducted in subjects without obesity at baseline and adjusted for TPE and BW at baseline and other co-variables. The results show significant reduction in the incidence of obesity (odds ratio (OR) = 0.095, 95% confidence interval (CI) 0.018 to 0.498; *p*-trend, 0.018) at the end of the follow up after adjustments.

Table 6 shows the associations between TPE at the fifth year and changes in anthropometric parameters, analyzed by linear regression models. For total participants, inverse associations were found between changes in BW (β = −0.363; *p* = 0.024) and BMI (β = −0.145; *p* = 0.023) and TPE in the fifth year after adjustment. For males, there was not any inverse association, while for females, inverse associations were found for changes in BW (β = −0.568; *p* = 0.008) and BMI (β = −0.221; *p* = 0.017) after adjustment.

Changes in obesity parameters for male, female, and total participants between baseline and end of follow up were observed (Table S1). For total participants, subjects in the highest TPE category had the lowest BW (70.29 ± 10.25 kg) and BMI (28.40 ± 3.75 kg/m^2^) after the intervention. Inversely, those participants in the first quintile of TPE had significantly higher WC (101.41 ± 9.35 cm) and WHtR (61.80 ± 5.15) compared with baseline values. For males, there was a significant inverse trend among quintiles and BW and BMI both at baseline and the fifth year. Also a significant reduction was observed comparing the top quintile with the bottom quintile both at baseline and the fifth year. For females, there was a significant reduction of BW and BMI in the top quintile groups after five years of intervention.

Table S2 shows the associations between changes in anthropometric parameters and changes in TPE over five years with linear regression models. For total participants, inverse associations were found between changes in BW (β = −0.355; *p* = 0.036), BMI (β = −0.139; *p* = 0.037) and TPE five years after adjustment. For males, there was not any inverse association, while, for females, inverse associations were found for changes in BW (β = −0.723; *p* = 0.003), BMI (β = −0.283; *p* = 0.006), and WC (β = −0.701; *p* = 0.046) after adjustment.

## 4. Discussion

In this five years study conducted in elderly participants at high cardiovascular risk, a higher total polyphenol intake, expressed as TPE, was inversely associated with weight parameters including BW, BMI, WC, and WHtR, as well as with the prevalence of obesity after a five years follow up, suggesting that polyphenols could be considered an independent contributor to the weight loss effects of a Mediterranean diet.

Several PREDIMED sub-trials have reported a range of mechanisms for the weight loss effects of a Mediterranean diet, including a high ingestion of dietary fiber, antioxidants, unsaturated fatty acids, extra virgin olive oil, nuts, and moderate wine consumption [29,30,31,32,33,34,35]. The reduction we observed in weight parameters might be partly attributed to the intake of the aforementioned food items; however, in the fully adjusted models, we removed their effects by adjusting for adherence to the Mediterranean diet (14 unit MedDiet questionnaire). Furthermore, even though the intake of these foods increased after five years of follow up, none of them showed significant differences within quintile categories at the end of the intervention; therefore, polyphenol intake could be considered an independent factor.

The present findings are consistent with previous reports on the inverse associations between polyphenol intake and weight parameters. A 16 years longitudinal study from the Netherlands associated a higher intake of total flavonols/flavones and catechins with a lower increase in BMI [36]. Other supporting evidence showed a significant decrease of 1.9 cm in WC and 1.2 kg in BW after supplementation of catechin-rich green tea for 90 days, although at a much higher dose than habitual intakes [37]. Two 12 week intervention studies also demonstrated the anti-obesity effects of green tea intake, finding a considerable reduction in BW, BMI, WC, and total abdominal fat area [38,39]. Another clinical trial indicated that consumption of normal or high-polyphenolic orange juice reduced body weight in obese or overweight adults, demonstrating an inverse association between polyphenol intake and body weight [10]. On the contrary, the effect of daily decaffeinated green tea intake on weight and body composition were tested among a group population in overweight breast cancer survivors. Results showed a slight but not significant increasing in weight loss after the intervention [40]. Compared with Asian populations, Caucasians show inconsistent results: a study showed no effects on body weight with long-term green tea extract supplementation [11]; another study with relapsing-remitting multiple sclerosis patients using one of the main green tea polyphenols, (-)epigal-locatechin-3-gallate, after three months of consumption showed greater muscle metabolism improvement in males than females [41]; supplementation with resveratrol exerted significant effects on energy metabolism in obese subjects, while another two findings showed ineffectiveness in nonobese women and obese men [42,43,44]. The results are inconsistent probably because of the different doses of polyphenol intakes, sex-specific effects, sample sizes, or length of duration.

The Mediterranean diet could be considered rich in polyphenol content because it is characterized by a high consumption of fruit and vegetables, virgin olive oil, legumes, and nuts and a moderate consumption of wine [45]. Results from a meta-analysis of 16 randomized controlled trials with a Mediterranean diet showed an average reduction in participant weight of 1.75 kg and a reduction in BMI of 0.57 kg/m^2^, as well as a greater reduction in BW of 3.88 kg under conditions of energy restriction, suggesting that adherence to a Mediterranean diet helps to control weight [46]. The PREDIMED, the European Prospective Investigation into Cancer and Nutrition (EPIC-Spain) cohort and the Seguimiento Universidad de Navarra (SUN) cohort, also in Spain, have shown in the long-term a significantly lower risk of overweightness/obesity associated with better Mediterranean diet adherence [34,47,48]. We observed a 1.22 kg decrease in BW and 0.50 in BMI in the highest TPE quintile, which partly agrees with previous studies reporting a similar reduction in body weight parameters.

Indexes of abdominal obesity, namely WC and WHtR, were significantly lower in the highest TPE quintile. These parameters are more accurate discriminators of cardiovascular risk than BMI due to the closer relationship between cardiovascular disease and abdominal obesity [49]. In agreement with our findings, in a PREDIMED study, and several other studies, the Mediterranean diet was negatively associated with WC and WHtR [50,51,52]. Additionally, two feeding trials with green tea polyphenol extracts also showed beneficial effects on abdominal obesity parameters [39,53].

Our results indicate the weight loss effect of polyphenols is higher in females than males. There are several possible reasons to explain the observed results: first, the prevalence of obesity is higher in women than men, and weight loss tends to be lower in obese individuals [54]. In the current study, at baseline, the prevalence of obesity was 32.9% for males and 44.9% for females, which is in line with another elderly population in United States [55]. Second, a higher TPE increment was observed when comparing females and males after five years, which may potentially explain the difference in weight loss effectiveness. Third, self-characteristics concerning males and females may also contribute to the difference. Women tend to have higher concentrations of leptin, an appetite regulation hormone that helps to reduce energy intake [56]. Evidence shows a significant inverse association between polyphenol intakes and plasma leptin levels, indicating higher polyphenol intake responses to better weight loss effectiveness [10]. Furthermore, individual differences in the composition of the gut microbiota may also contribute to differences in bioavailability and polyphenolic metabolites, further influencing the weight loss effectiveness [57].

We also found less gain in WC in males, after five years of intervention. The observation could be primarily explained by the greater percentage of muscle mass and mineral mass in males compared with females. Waist circumference is affected by age, body weight, body composition, and fat distribution [58]. Various types of polyphenol help to reduce visceral fat [9]. Additionally, since waist circumference increases with age, it is worth noting that the average age is older in females in our population, which could be considered another possible explanation.

Potential explanations of the observed inverse association between polyphenol intake and weight loss likely involve several mechanisms due to the diversity of polyphenol chemical structures, complex metabolic pathways, and oral bioavailability. Excess adipose mass and adipose tissue expansion results from adipocyte hypertrophy and hyperplasia [59]. Common plausible mechanisms include the suppression of fat absorption and anabolic pathways; inhibition of adipogenesis and lipogenesis; stimulation of catabolic pathways with increment of lipolysis, apoptosis of mature adipocytes, and acid β-oxidation; reduction of chronic inflammatory response relative to adiposity; and increment in energy expenditure through up-regulating uncoupling protein (UCP1-3) [8,9]. However, knowledge of the anti-obesity effects of polyphenols is limited and only a few specific compounds have been analyzed in this context. For instance, it has been demonstrated that resveratrol, widely present in red grapes and red wine, exerts an anti-obesity action by reducing adipogenesis and increasing apoptosis in mature adipocytes and inhibiting fat accumulation processes and stimulating lipolytic and oxidative pathways in vivo studies and clinical trials [43,60,61]. Contrary results have been observed with anthocyanins, water-soluble plant pigments in blue, purple, and red fruits. On one hand, they seem to significantly reduce body weight. This effect may be due to the suppression of lipid synthesis, the up-regulation of adiponectin, which enhances insulin sensitivity, and the reduction in of serum triglycerides and leptin levels [8,62]. However, two clinical trials showed non-significant reduction trends in body weight after supplementation with food rich in anthocyanins [63,64]. The anti-obesity effects of flavonoids, which are a large group of polyphenols found in a wide range of Mediterranean diet foods [65,66], have been mainly attributed to improvement in adipocyte functionality and fat oxidation [67]. Also playing a key role in weight control is the down-regulation of a variety of pro-inflammatory adipocytokines, particularly tumor necrosis factor alpha (TNF-α) [68]. A clinical trial indicates that the inhibition of intestinal fat absorption may contribute to the weight loss after an ingestion of a green tea beverage enriched with catechins [69]. In summary, even though the intake of some specific polyphenols has been associated with body weight management, there is still not enough evidence for the effect of total polyphenols or some classes of polyphenols, and further studies are needed to explore the mechanisms involved as well as potential synergistic effects among them.

The association between weight loss and improvement in cardiovascular risk factors has been widely discussed. Numerous studies indicate that polyphenol intake reduces cardiovascular risk factors [70,71]. A previous study found a significant improvement in cardiovascular risk factors, with a 5–10% of weight loss after one year [72]. We also found protective effects on cardiovascular risk factors, including diastolic blood pressure, glucose concentration, and triglycerides concentration, with the same population after five years of intervention [73]. A randomized study conducted in healthy participants, feed with apple polyphenol, also supported that an improvement in cardiovascular risk factor helps to regulate fat metabolism [74].

Some limitations of this study should be noted. First, given that the study was conducted among elderly subjects at high cardiovascular risk, the results cannot be extrapolated to the general population. Second, even though we adjusted for major potential confounders, we still cannot exclude residual confounding from measurements. Third, even though WC and WHtR may reflect abdominal obesity more accurately, they cannot differentiate between fat distribution in visceral adipose tissue and subcutaneous abdominal adipose tissue; hence we cannot conclude if a reduction in abdominal obesity parameters is beneficial to visceral or subcutaneous fat mass or both [75]. Another limitation is the lack of specific measurements of polyphenol metabolism in vivo. 

The present study also has several strengths. Its main strong point is the use of TPE, a biomarker of polyphenol intake, which could provide more precise data than measuring total polyphenol intake through self-reported information in FFQs or databases. Another strength is its prospective design. Only a few studies have analyzed the association between total polyphenol intake and weight control, and the current work is the first to associate anti-obesity effects with total polyphenol intake in individuals at high cardiovascular risk [8,76]. In addition, the long-term duration of the intervention provides more robust results compared with other short-term trials.

## 5. Conclusions

In summary, with five years of follow up, the present study shows that polyphenol levels expressed as TPE in urine were inversely associated with BW, BMI, WC, and WHtR in an elderly population at high cardiovascular risk. Therefore, we confirmed that a long-term polyphenol-rich diet contributes to body weight loss, which can offer protection from several chronic diseases. For future research, similar studies should be conducted in the general population, and specific mechanisms need to be explored by further clinical trials.

## Figures and Tables

**Table 1:** Baseline characteristics of participants according to quintiles of total urinary polyphenol excretion (TPE) at baseline.

	TPE (mg GAE/g Creatinine)										
	Q1		Q2		Q3		Q4		Q5		*p*
	(<76.55)		(76.56–95.20)		(95.21–119.18)		(119.19–145.86)		(>145.86)		
No. of subjects	114	-	115	-	115	-	115	-	114	-	-
Women, *n* (%)	33	28	49	42.6	66	57.4	65	56.5	83	72.8	<0.001
Age (y), mean (SD)	66.4	5.9	66.6	6.0	66.9	5.9	67.7	5.7	68.9	5.8	0.007
Weight (kg), mean (SD)	80.2	11.8	77.7	10.4	73.7	9.1	73.3	10.9	70.8	10.4	<0.001
BMI (kg/m^2^), mean (SD)	29.8	2.9	29.5	3.2	29.1	3.1	28.9	3.6	28.8	3.4	0.080
Systolic BP (mm Hg), mean (SD)	150.9	16.9	153.9	19.5	149.9	17.0	151.8	19.5	150.4	15.9	0.454
Diastolic BP (mm Hg), mean (SD)	86.4	10.5	86.1	10.4	85.0	9.9	84.2	10.3	84.4	9.1	0.363
Hypertension, *n* (%)	93	81.6	102	88.7	94	81.7	79	68.7	93	81.6	0.004
Diabetes, *n* (%)	47	41.2	41	35.7	48	41.7	53	46.1	49	43.0	0.605
Dyslipidemia, *n* (%)	72	63.2	74	64.3	78	67.8	80	69.6	79	69.3	0.779
Smoking status, *n* (%)											0.002
Current	32	28.1	22	19.1	16	13.9	18	15.7	9	7.9	-
Former	26	22.8	25	21.7	24	20.9	31	27.0	20	17.5	-
Never	56	49.1	68	59.1	75	65.2	66	57.4	85	74.6	-
Family history of CHD, *n* (%)	39	34.2	42	36.5	45	39.1	41	35.7	48	42.1	0.95
Medication, *n* (%)											-
Aspirin	21	18.4	17	14.8	27	23.5	19	16.5	19	16.7	0.482
Antihypertensive drugs	87	76.3	90	78.3	82	71.3	71	61.7	84	73.7	0.049
Hypolipidemic drugs	40	35.1	45	39.1	50	43.5	53	46.1	51	44.7	0.426
Insulin	3	2.6	7	6.1	4	3.5	7	6.1	6	5.3	0.639
Oral hypoglycemic drugs	23	20.2	22	19.1	29	25.2	30	26.1	27	23.7	0.652
Vitamin or minerals	5	4.4	5	4.3	10	8.7	9	7.8	18	15.8	0.005
Education level, *n* (%)											0.348
University	13	11.4	16	13.9	10	8.7	7	6.1	9	7.9	-
High school	21	18.4	14	12.2	18	15.7	22	19.1	13	11.4	-
Primary school	79	69.3	83	72.2	85	73.9	86	74.8	92	80.7	-
Marital status, *n* (%)											0.168
Single	7	6.1	5	4.3	4	3.5	3	2.6	6	5.3	-
Married	96	84.2	93	80.9	93	80.9	88	76.5	80	70.2	-
Widowed	10	8.8	14	12.2	16	13.9	24	20.9	25	21.9	-
Physical activity at leisure time (MET-min/d), mean (SD)	267.5	222.5	302.7	256.3	233	172.9	283.7	271.3	261.7	247.3	0.237

TPE: total polyphenol excretion; GAE: gallic acid equivalent; BMI: body mass index; BP: blood pressure; CHD: coronary heart diseases. Data are given as means (SD) for continuous variables and percentages for categorical variables; *p* < 0.05 indicates statistical significance. * *p*-values calculated by analysis of variance or χ^2^ tests.

**Table 2:** Changes in key food intake and nutrients according to the food frequency questionnaires (FFQs) after energy adjustment categorized by quintile of TPE at the fifth year ^a^.

		TPE (mg GAE/g Creatinine)													
		Q1		Q2		Q3		Q4		Q5		*p* ^b^	*p* ^c^		
		(<79.02)		(79.03–99.50)		(99.51–124.53)		(124.54–160.06)		(>160.07)					
		Mean	SD	Mean	SD	Mean	SD	Mean	SD	Mean	SD	ANOVA	TIME	GROUP	INTERACTION
Vegetables (g/day)	baseline	302.6	126.5	295.2	122.6	283.3	114.1	312.9	142.4	297.0	107.0	0.484	<0.001	0.916	0.440
	changes	47.7 **	131.5	53.1 **	130.2	76.3 **	150.4	46.0 **	162.3	59.0 **	131.6	0.514			
Fruits (g/day)	baseline	328.9	184.8	361.6	179.7	358.6	182.3	389.5	162.5	394.5	189.3	0.064	<0.001	0.051	0.530
	changes	89.1 **	220.1	82.9 **	222.7	111.0 **	220.6	80.6 **	208.4	67.9 **	194.8	0.658			
Legumes (g/day)	baseline	18.5	8.4	19.3	7.4	20.0	9.2	19.2	7.2	19.0	6.6	0.634	0.446	0.251	0.045
	changes	0.3	10.7	1.7	11.3	−3.2	29.1	−1.0	8.5	1.0	8.3	0.160			
Cereals (g/day)	baseline	246.8	83.8	246.3	81.2	232.8	74.2	242.9	71.9	237.7	61.9	0.636	<0.001	0.530	0.386
	changes	−22.2 *	91.1	−21.7 **	85.1	−14.5	91.5	−31.4 **	80.1	−25.6 **	77.5	0.673			
Milk (g/day)	baseline	322.8	191.3	370.1	202.2	365.8	210.0	354.5	203.7	422.5	231.6	0.010	0.005	0.009	0.728
	changes	38.3	207.4	21.4	200.9	10.3	198.6	40.2 *	188.3	20.6	208.9	0.776			
Meat (g/day)	baseline	138.1	50.4	136.2	43.9	145.6	56.5	139.3	41.7	141.6	47.1	0.613	<0.001	0.198	0.983
	changes	−9.9	52.1	−13.9 **	44.6	−12.1 *	52.5	−13.8 **	51.5	−11.7 *	47.7	0.975			
Fish (g/day)	baseline	92.0	39.6	92.5	36.9	90.4	38.0	97.0	41.3	93.0	39.0	0.787	0.005	0.970	0.481
	changes	8.2 *	40.4	6.2	42.2	11.5 **	44.2	4.2	39.4	7.3	43.3	0.764			
Pastries (g/day)	baseline	29.6	32.1	23.6	22.4	24.1	24.7	23.9	22.4	26.6	26.5	0.379	0.006	0.291	0.920
	changes	−4.8	32.4	−3.0	29.4	−4.3	27.2	−3.2	24.3	−3.9	29.2	0.991			
EVOO (g/day)	baseline	22.7	25.6	20.5	22.4	22.6	23.6	22.9	23.7	22.8	23.3	0.960	<0.001	0.961	0.626
	changes	24.9 **	29.6	27.6 **	27.6	25.4 **	28.2	26.4 **	30.1	26.9 **	25.6	0.955			
Olive oil (g/day)	baseline	45.4	17.6	46.3	14.2	45.3	13.5	46.3	14.5	44.2	15.3	0.791	<0.001	0.575	0.161
	changes	7.8 **	18.2	10.5 **	17.5	9.8 **	16.8	9.3 **	18.7	11.2 **	16.8	0.654			
Nuts (g/day)	baseline	10.7	14.2	11.2	12.6	10.0	12.7	10.0	13.6	10.9	11.5	0.904	<0.001	0.634	0.192
	changes	3.1	16.4	5.3 **	17.1	5.5 **	15.8	9.5 **	17.3	4.2 **	14.0	0.039			
Wine (g/day)	baseline	104.9	144.8	97.0	138.3	103.9	171.5	98.2	136.2	81.2	125.6	0.739	0.013	0.647	0.984
	changes	−8.4	124.2	−13.3	129.4	−17.1	111.3	−18.1 *	95.3	−14.5	91.9	0.971			
Tea (mL)	baseline	4.8	14.5	4.6	15.1	6.4	17.0	5.2	12.5	7.6	21.1	0.605	0.401	0.479	0.172
	changes	0.1	16.6	−1.9	14.5	−1.8	16.7	3.2	24.9	−2.0	22.4	0.204			
Coffee (mL)	baseline	39.1	58.4	36.7	52.4	30.7	43.0	35.0	47.1	30.9	43.0	0.717	0.002	0.546	0.098
	changes	−11.5 *	50.1	−1.8	50.2	−7.3 *	36.8	−13.1 **	47.0	3.7	51.3	0.048			
Total carbohydrates (g/day)	baseline	237.5	43.2	242.1	38.0	237.0	41.7	235.8	36.3	239.7	34.8	0.682	0.769	0.114	0.025
	changes	4.5	78.6	−10.3	71.6	5.7	72.4	−13.2 *	68.1	−0.4	64.6	0.166			
Protein (g/day)	baseline	90.3	43.2	94.9	38.0	89.7	41.7	88.5	36.3	92.5	34.8	0.682	0.274	0.307	0.474
	changes	2.3	47.6	−3.0	41.4	6.1	46.9	2.5	40.9	4.6	39.6	0.592			
Total Fat (g/day)	baseline	100.4	13.6	100.9	12.7	102.8	13.7	102.7	12.9	104.5	13.6	0.132	<0.001	0.235	0.981
	changes	10.0 **	30.8	9.2 **	28.5	10.9 **	28.0	8.5 **	26.9	9.4 **	31.5	0.980			
Fiber (g/day)	baseline	24.2	6.0	24.5	6.5	24.2	6.5	25.8	5.8	25.5	5.6	0.256	0.006	0.632	0.013
	changes	1.1	8.2	1.4	9.7	3.2 **	8.4	0.2	8.2	1.0	7.8	0.107			
Alcohol (g/day)	baseline	13.5	5.6	13.6	5.2	13.7	5.3	13.3	4.7	14.2	4.9	0.780	0.032	0.961	0.765
	changes	−0.2	17.8	−1.6	15.0	−1.2	16.7	−1.9	14.4	−3.2 *	14.2	0.707			
SFA (g/day)	baseline	24.9	11.2	24.2	9.7	24.5	8.3	25.7	9.1	23.9	9.9	0.663	0.541	0.772	0.266
	changes	−0.5	12.9	−0.1	10.6	0.7	9.8	−1.5	11.1	1.6	11.3	0.301			
MUFA (g/day)	baseline	52.1	16.8	51.3	15.4	52.4	16.0	53.6	14.5	52.4	15.0	0.828	<0.001	0.941	0.694
	changes	6.0 **	20.3	7.5 **	19.2	7.4 **	17.5	4.8 **	18.1	7.1 **	19.0	0.798			
PUFA (g/day)	baseline	15.6	5.2	15.4	5.7	15.5	5.8	16.0	5.2	15.5	5.5	0.887	<0.001	0.575	0.670
	changes	3.0 **	7.7	3.1 **	7.8	3.4 **	7.1	3.4 **	8.1	4.2 **	8.9	0.800			
Folic acid (μg/day)	baseline	379.3	89.5	373.3	90.5	369.8	76.5	394.4	98.8	394.2	85.7	0.152	<0.001	0.447	0.086
	changes	42.7 **	91.3	46.5 **	99.7	65.7 **	92.6	39.2 **	91.6	38.7 **	100.4	0.196			
Cholesterol (mg/day)	baseline	354.9	92.4	340.0	92.2	351.4	93.3	353.5	81.6	359.1	95.5	0.505	0.234	0.406	0.329
	changes	0.2	115.0	11.9	101.3	11.4	109.2	−3.5	105.5	15.2	112.6	0.644			
Na (mg/day)	baseline	2331.2	570.5	2254.1	499.7	2272.1	480.3	2286.1	491.3	2298.5	447.1	0.815	0.257	0.258	0.319
	changes	−64.0	941.6	−83.1	728.6	10.6	730.0	−184.2 **	713.4	10.8	752.6	0.306			
K (mg/day)	baseline	4130.5	769.6	4218.7	756.2	4208.9	696.0	4312.6	808.1	4410.2	748.0	0.069	<0.001	0.246	0.067
	changes	379.8 **	1070.7	286.0 **	1042.2	497.3 **	1007.2	249.1 *	1116.2	268.1 *	1100.4	0.389			
Mg (mg/day)	baseline	355.0	64.0	361.1	68.6	356.	56.6	368.6	58.2	374.8	61.9	0.147	<0.001	0.474	0.056
	changes	30.7 **	97.9	26.8 **	99.2	42.9 **	87.9	22.0 **	87.3	23.9 *	95.9	0.481			
P-14 score	baseline	8.9	1.8	9.0	1.8	9.2	1.9	8.8	1.9	9.1	1.8	0.441	<0.001	0.370	0.571
	changes	1.6 **	2.5	1.8 **	1.9	1.7 **	2.1	1.9 **	2.0	1.6 **	2.1	0.626			
Energy intake (Kcal/day)	baseline	2508.8	582.3	2285.5	578.5	2338.5	463.0	2262.2	510.7	2191.7	470.7	<0.001	0.034	<0.001	0.234
	changes	−25.9	627.6	32.0	497.4	100.2 *	503.9	8.9	564.0	157.8 **	520.1	0.089			

TPE: total polyphenol excretion; GAE: gallic acid equivalent; EVOO: Extra Virgin Olive Oil; SFA: saturated fatty acids; MUFA: monounsaturated fatty acids; PUFA: polyunsaturated fatty acids; Na: sodium; K: potassium; Mg: magnesium; *p*-14: 14 item dietary score test to appraise adherence of participants to the Mediterranean diet (it includes questions about consumption of fruits, vegetables, meat, fish, carbonic beverages, legumes, nuts, olive oil, wine, and culinary methods) [28]. ^a^ Data are given as means (SD); *p* < 0.05 indicates statistical significance. Values with asterisks are statistically different from baseline by paired-samples *t*-test (* *p* < 0.05; ** *p* < 0.01). ^b^ Data were analyzed by one-way ANOVA. ^c^ Data analyzed by repeated-measures two-factor ANOVA.

**Table 3:** Multiple linear regression analyses with obesity indexes and quintiles of TPE at the fifth year for male, female, and total participants.

			β	SE	Beta	Significance	95% CI	
BW (kg)	Male	Model 1	−1.446	0.440	−0.195	0.001	−2.313	−0.580
		Model 2	−1.259	0.440	−0.170	0.005	−2.126	−0.392
		Model 3	−0.959	0.461	−0.131	0.039	−1.868	−0.050
	Female	Model 1	−1.103	0.415	−0.153	0.008	−1.920	−0.287
		Model 2	−0.756	0.414	−0.105	0.069	−1.571	0.058
		Model 3	−0.757	0.431	−0.107	0.080	−1.606	0.091
	Total	Model 1	−2.350	0.331	−0.285	<0.001	−3.000	−1.700
		Model 2	−1.070	0.315	−0.130	0.001	−1.689	−0.451
		Model 3	−1.004	0.320	−0.124	0.002	−1.634	−0.375
BMI (kg/m^2^)	Male	Model 1	−0.405	0.135	−0.179	0.003	−0.670	−0.139
		Model 2	−0.370	0.136	−0.164	0.007	−0.639	−0.102
		Model 3	−0.301	0.141	−0.135	0.034	−0.579	−0.023
	Female	Model 1	−0.344	0.156	−0.127	0.028	−0.652	−0.037
		Model 2	−0.296	0.160	−0.110	0.064	−0.611	0.018
		Model 3	−0.332	0.165	−0.123	0.046	−0.657	−0.007
	Total	Model 1	−0.295	0.104	−0.118	0.005	−0.499	−0.090
		Model 2	−0.328	0.110	−0.131	0.003	−0.544	−0.111
		Model 3	−0.320	0.113	−0.129	0.005	−0.541	−0.098
WC (cm)	Male	Model 1	−0.769	0.364	−0.127	0.036	−1.487	−0.052
		Model 2	−0.786	0.369	−0.130	0.034	−1.513	−0.059
		Model 3	−0.516	0.378	−0.087	0.173	−1.260	0.228
	Female	Model 1	−0.546	0.409	−0.078	0.183	−1.351	0.259
		Model 2	−0.527	0.419	−0.075	0.209	−1.351	0.297
		Model 3	−0.701	0.434	−0.101	0.108	−1.556	0.154
	Total	Model 1	−1.500	0.296	−0.208	<0.001	−2.082	−0.918
		Model 2	−0.721	0.293	−0.100	0.014	−1.296	−0.147
		Model 3	−0.742	0.297	−0.104	0.013	−1.326	−0.158
WHtR (cm/m)	Male	Model 1	−0.340	0.220	−0.093	0.124	−0.773	0.094
		Model 2	−0.385	0.223	−0.105	0.085	−0.823	0.054
		Model 3	−0.258	0.229	−0.072	0.261	−0.710	0.193
	Female	Model 1	−0.246	0.276	−0.052	0.374	−0.789	0.298
		Model 2	−0.332	0.282	−0.070	0.240	−0.887	0.223
		Model3	−0.501	0.294	−0.106	0.089	−1.079	0.078
	Total	Model 1	−0.298	0.178	−0.070	0.094	−0.648	0.051
		Model 2	−0.367	0.189	−0.087	0.052	−0.739	0.004
		Model 3	−0.408	0.194	−0.097	0.036	−0.788	−0.028

BW: body weight; BMI: body mass index; WC: waist circumference; WHtR: waist-to-height ratio. TPE: total polyphenol excretion (mg GAE/g creatinine). β: Non-standardized coefficient (regression line coefficient); SE: Standard error; Beta: Standardized coefficient; CI: Confidence interval; *p*: two-sided test of significance. Model 1, unadjusted; Model 2 was adjusted for sex (only for total participants), age, and intervention groups; Model 3 adjusted as in Model 2 plus smoking status (never, current, former), family history of CHD, physical activity, hypertension, diabetes, dyslipidemia, marital status (single, married, divorced, widowed), education level (primary school, high school, university), medication used (antihypertensive drugs, vitamins, insulin, oral hypoglycemic drugs, aspirin, or other antiplatelet drug supplements taken in the last month), recruitment centers, 14 unit Mediterranean diet score, and energy intake at baseline.

**Table 4:** Multivariate adjusted odds ratios (95% confidence interval (CI)) for prevalent obesity (213 cases) after a five years follow up.

		Q1	Q2	95% CI		Q3	95% CI		Q4	95% CI		Q5	95% CI		*p*
Male	Model 1	1 (ref.)	0.531	0.244	1.155	0.545	0.250	1.188	0.488	0.223	1.070	0.313	0.135	0.722	0.095
(90 case)	Model 2	1 (ref.)	0.559	0.254	1.229	0.571	0.260	1.254	0.520	0.235	1.148	0.340	0.146	0.792	0.159
	Model 3	1 (ref.)	0.586	0.243	1.416	0.588	0.238	1.452	0.511	0.204	1.283	0.387	0.146	1.029	0.418
Female	Model 1	1 (ref.)	0.934	0.454	1.924	0.791	0.384	1.628	0.643	0.310	1.333	0.429	0.200	0.919	0.195
(123 case)	Model 2	1 (ref.)	1.041	0.497	2.182	0.844	0.403	1.768	0.769	0.361	1.638	0.493	0.226	1.078	0.352
	Model 3	1 (ref.)	1.257	0.538	2.934	0.748	0.317	1.764	0.595	0.244	1.450	0.461	0.181	1.170	0.223
Total	Model 1	1 (ref.)	0.639	0.375	1.089	0.769	0.454	1.302	0.664	0.390	1.129	0.450	0.259	0.782	0.073
(213 case)	Model 2	1 (ref.)	0.597	0.344	1.035	0.691	0.400	1.192	0.618	0.350	1.091	0.383	0.211	0.694	0.036
	Model 3	1 (ref.)	0.604	0.332	1.100	0.720	0.399	1.300	0.560	0.298	1.054	0.346	0.176	0.678	0.039

Quintiles for males: Q1 < 70.61; Q2: 70.62–88.94; Q3: 88.95–108.61; Q4: 108.62–137.11; Q5 > 137.11; Quintiles for females: Q1 < 91.67; Q2: 91.68–113.96; Q3: 113.97–138.28; Q4: 138.29–181.01; Q5 > 181.01; Quintiles for total: Q1 < 79.02; Q2: 79.03–99.50; Q3: 99.51–124.53; Q4: 124.54–160.06; Q5 > 160.06. TPE is expressed as mg GAE/g creatinine. Obesity was defined as BMI > 30 kg/m^2^. Model 1, unadjusted; Model 2 was adjusted for sex (only for total participants), age, and intervention groups; Model 3 adjusted as in Model 2 plus smoking status (never, current, former), family history of CHD, physical activity, hypertension, diabetes, dyslipidemia, marital status (single, married, divorced, widowed), education level (primary school, high school, university), medication used (antihypertensive drugs, vitamins, insulin, oral hypoglycemic drugs, aspirin or other antiplatelet drug supplements taken in the last month) recruitment centers, 14 unit Mediterranean diet score, and energy intake at baseline.

**Table 5:** Association between TPE after five years of follow up and the incidence of obesity (39 new-onset case).

	Q1	Q2	95% CI		Q3	95% CI		Q4	95% CI		Q5	95% CI		*p*
Model 1	1 (ref.)	0.912	(0.337	2.468)	0.676	(0.253	1.810)	0.351	(0.142	0.866)	0.406	(0.164	1.005)	0.054
Model 2	1 (ref.)	0.454	(0.185	1.115)	0.235	(0.072	0.767)	0.285	(0.093	0.868)	0.145	(0.037	0.558)	0.014
Model 3	1 (ref.)	0.382	(0.146	1.001)	0.193	(0.055	0.676)	0.272	(0.084	0.885)	0.119	(0.028	0.505)	0.014
Model 4	1 (ref.)	0.366	(0.126	1.062)	0.156	(0.040	0.612)	0.218	(0.054	0.881)	0.095	(0.018	0.498)	0.018

Model 1, unadjusted; Model 2 was adjusted for baseline TPE and baseline BW; Model 3 was adjusted as in Model 2 plus sex, age, and intervention groups; Model 4 was adjusted as in Model 3 plus smoking status (never, current, former), family history of CHD, physical activity, hypertension, diabetes, dyslipidemia, marital status (single, married, divorced, widowed), education level (primary school, high school, university), medication used (antihypertensive drugs, vitamins, insulin, oral hypoglycemic drugs, aspirin, or other antiplatelet drug supplements taken in the last month) recruitment centers, 14 unit Mediterranean diet score, and energy intake at baseline.

**Table 6:** Multiple linear regression analyses with changes in anthropometric parameters and quintiles of TPE at the fifth year.

			β	SE	Beta	*p*	95% CI	
Changes in BW	Male	Model 1	−0.098	0.211	−0.028	0.642	−0.514	0.318
(kg)		Model 2	−0.229	0.217	−0.066	0.294	−0.657	0.199
		Model 3	−0.186	0.217	−0.053	0.393	−0.614	0.242
		Model 4	−0.037	0.233	−0.011	0.872	−0.495	0.421
	Female	Model 1	−0.648	0.193	−0.193	0.001	−1.027	−0.269
		Model 2	−0.664	0.196	−0.197	0.001	−1.049	−0.279
		Model 3	−0.573	0.197	−0.17	0.004	−0.961	−0.185
		Model 4	−0.568	0.213	−0.169	0.008	−0.987	−0.149
	Total	Model 1	−0.429	0.142	−0.125	0.003	−0.709	−0.149
		Model 2	−0.539	0.15	−0.157	<0.001	−0.835	−0.244
		Model 3	−0.436	0.153	−0.127	0.005	−0.737	−0.135
		Model 4	−0.363	0.161	−0.108	0.024	−0.68	−0.047
Changes in BMI	Male	Model 1	−0.04	0.075	−0.033	0.589	−0.187	0.107
(kg/m^2^)		Model 2	−0.087	0.077	−0.07	0.259	−0.238	0.064
		Model 3	−0.072	0.077	−0.059	0.348	−0.224	0.079
		Model 4	−0.019	0.082	−0.016	0.817	−0.18	0.142
	Female	Model 1	−0.256	0.083	−0.177	0.002	−0.419	−0.092
		Model 2	−0.262	0.084	−0.181	0.002	−0.428	−0.096
		Model 3	−0.223	0.085	−0.154	0.009	−0.391	−0.056
		Model 4	−0.221	0.092	−0.153	0.017	−0.402	−0.04
	Total	Model 1	−0.176	0.056	−0.13	0.002	−0.286	−0.065
		Model 2	−0.215	0.059	−0.159	<0.001	−0.331	−0.098
		Model 3	−0.172	0.06	−0.128	0.005	−0.291	−0.054
		Model 4	−0.145	0.064	−0.109	0.023	−0.27	−0.02
Changes in WC	Male	Model 1	−0.066	0.233	−0.017	0.776	−0.525	0.392
(cm)		Model 2	−0.209	0.241	−0.054	0.386	−0.684	0.265
		Model 3	−0.17	0.242	−0.044	0.484	−0.647	0.307
		Model 4	−0.108	0.249	−0.029	0.666	−0.599	0.383
	Female	Model 1	−0.34	0.275	−0.072	0.217	−0.88	0.2
		Model 2	−0.325	0.28	−0.069	0.247	−0.877	0.227
		Model 3	−0.31	0.286	−0.066	0.279	−0.872	0.253
		Model 4	−0.42	0.311	−0.089	0.178	−1.033	0.192
	Total	Model 1	−0.237	0.181	−0.055	0.190	−0.592	0.118
		Model 2	−0.302	0.192	−0.070	0.118	−0.680	0.076
		Model 3	−0.252	0.198	−0.059	0.203	−0.640	0.136
		Model 4	−0.269	0.207	−0.063	0.195	−0.676	0.138
Changes in WHtR	Male	Model 1	−0.066	0.233	−0.017	0.776	−0.525	0.392
(cm/m)		Model 2	−0.209	0.241	−0.054	0.386	−0.684	0.265
		Model 3	−0.17	0.242	−0.044	0.484	−0.647	0.307
		Model 4	−0.108	0.249	−0.029	0.666	−0.599	0.383
	Female	Model 1	−0.202	0.179	−0.066	0.262	−0.555	0.152
		Model 2	−0.194	0.183	−0.063	0.29	−0.555	0.166
		Model 3	−0.184	0.187	−0.06	0.325	−0.552	0.183
		Model 4	−0.262	0.204	−0.085	0.199	−0.663	0.139
	Total	Model 1	−0.142	0.114	−0.052	0.216	−0.367	0.083
		Model 2	−0.184	0.122	−0.068	0.131	−0.424	0.055
		Model 3	−0.155	0.125	−0.057	0.215	−0.401	0.091
		Model 4	−0.167	0.131	−0.062	0.203	−0.426	0.091

TPE: total polyphenol excretion; GAE: gallic acid equivalent; BW: body weight; BMI: body mass index. WC: waist circumference; WHtR: waist-to-height ratio. Model 1, unadjusted; Model 2 was adjusted for baseline TPE and baseline BW; Model 3 was adjusted as in Model 2 plus sex (only for total participants), age, and intervention groups; Model 4 was adjusted as in Model 3 plus smoking status (never, current, former), family history of CHD, physical activity, hypertension, diabetes, dyslipidemia, marital status (single, married, divorced, widowed), education level (primary school, high school, university), medication used (antihypertensive drugs, vitamins, insulin, oral hypoglycemic drugs, aspirin, or other antiplatelet drug supplements taken in the last month) recruitment centers, 14 unit Mediterranean diet score, and energy intake at baseline.

## Supplementary Materials

The following are available online at www.mdpi.com/2072-6643/9/5/452/s1, Table S1: Comparisons of obesity indexes, Table S2: Multiple linear regression analyses with changes in anthropometric parameters and changes in quintiles of TPE over five years, File S1: STROBE checklist, File S2: Protocol of the PREDIMED study, File S3: Database of participants.
