# Supplementary material for: Polyphenol Levels Are Inversely Correlated with Body Weight and Obesity in an Elderly Population after 5 Years of Follow Up (The Randomised PREDIMED Study)
Source: Nutrients. 2017 May 3;9(5):452. doi: 10.3390/nu9050452 (PMC5452182; doi:10.3390/nu9050452)
Supplement: Supplementary file 1 [file nutrients-09-00452-s001.zip › Supplementary Materials/Table S1. Comparisons of obesity indexes.docx]

**Table S1. Comparisons of obesity indexes ^a^**

|  |  |  | Q1 | | Q2 | | Q3 | | Q4 | | Q5 | | P ^b^ | P ^c^ | P ^d^ |
| --- | --- | --- | --- | --- | --- | --- | --- | --- | --- | --- | --- | --- | --- | --- | --- |
|  |  |  | Mean | SD | Mean | SD | Mean | SD | Mean | SD | Mean | SD | Q1VsQ5 | ANOVA |  |
| BW (kg) | Male | Baseline | 83.54 | 9.70 | 82.44 | 9.84 | 79.23 | 9.25 | 79.80 | 11.05 | 77.82 | 9.30 | 0.026 | 0.014 |  |
|  |  | 5^th^-year | 83.96 | 9.52 | 81.37 | 10.16 | 79.39 | 10.13 | 79.50 | 11.73 | 77.63 | 9.77 | 0.016 | 0.021 | 0.616 |
|  |  | Changes | 0.72 | 4.74 | -1.08 | 5.11 | 0.16 | 4.70 | -0.31 | 6.14 | -0.19 | 3.50 | 1.000 | 0.418 |  |
|  | Female | Baseline | 69.78 | 9.70 | 72.22 | 8.84 | 69.94 | 9.98 | 69.31 | 9.77 | 68.95 | 8.52 | 1.000 | 0.361 |  |
|  |  | 5^th^-year | 70.87 | 10.59 | 72.20 | 9.93 | 68.57 | 10.22 | 69.17 | 10.51 | 66.86 | 8.91 | 0.317 | 0.044 | 0.07 |
|  |  | Changes | 1.10 | 5.71 | -0.01 | 3.61 | -1.38 ^**^ | 4.49 | -0.14 | 3.46 | -2.09 ^**^ | 5.52 | 0.002 | 0.002 |  |
|  | Total | Baseline | 79.98 | 11.52 | 75.24 | 10.07 | 76.42 | 11.55 | 72.50 | 10.44 | 71.52 | 9.50 | <0.001 | <0.001 |  |
|  |  | 5^th^-year | 80.50 | 11.13 | 75.07 | 11.10 | 76.04 | 11.72 | 71.92 | 11.29 | 70.29^**^ | 10.25 | <0.001 | <0.001 | 0.101 |
|  |  | Changes | 0.72 | 5.06 | -0.17 | 5.06 | -0.39 | 5.04 | -0.57 | 4.23 | -1.23^**^ | 4.57 | 0.024 | 0.045 |  |
| BMI(Kg/m^2^) | Male | Baseline | 29.74 | 2.77 | 29.34 | 2.68 | 28.32 | 2.72 | 28.90 | 2.96 | 28.08 | 3.02 | 0.024 | 0.012 |  |
|  |  | 5^th^-year | 29.99 | 3.13 | 28.96 | 2.89 | 28.36 | 2.90 | 28.82 | 3.46 | 28.02 | 3.30 | 0.013 | 0.017 | 0.702 |
|  |  | Changes | 0.30 | 1.74 | -0.38 | 1.78 | 0.04 | 1.61 | -0.09 | 2.16 | -0.06 | 1.28 | 1.000 | 0.374 |  |
|  | Female | Baseline | 29.38 | 3.43 | 29.92 | 3.56 | 29.45 | 3.49 | 29.80 | 3.85 | 28.99 | 3.48 | 1.000 | 0.638 |  |
|  |  | 5^th^-year | 29.79 | 3.44 | 29.89 | 3.73 | 28.87 | 3.60 | 29.76 | 4.30 | 28.12 | 3.75 | 0.173 | 0.048 | 0.062 |
|  |  | Changes | 0.41 | 2.62 | -0.04 | 1.51 | -0.58 ^**^ | 1.91 | -0.05 | 1.49 | -0.86 ^**^ | 2.27 | 0.006 | 0.006 |  |
|  | Total | Baseline | 29.53 | 2.92 | 29.07 | 3.07 | 29.44 | 3.41 | 29.09 | 3.43 | 28.90 | 3.44 | 1.000 | 0.549 |  |
|  |  | 5^th^-year | 29.81 | 3.04 | 28.98 | 3.31 | 29.30 | 3.63 | 28.85 | 3.76 | 28.40^**^ | 3.75 | 0.027 | 0.039 | 0.092 |
|  |  | Changes | 0.30 | 1.88 | -0.09 | 2.10 | -0.13 | 1.86 | -0.24 | 1.73 | -0.50^**^ | 1.87 | 0.015 | 0.031 |  |
| WC (cm) | Male | Baseline | 102.85 | 7.98 | 101.79 | 8.08 | 99.75 | 8.01 | 100.82 | 9.06 | 99.55 | 7.01 | 0.321 | 0.162 |  |
|  |  | 5^th^-year | 104.14 | 8.46 | 102.08 | 8.30 | 101.04 | 8.45 | 101.51 | 9.34 | 100.55 | 7.99 | 0.293 | 0.222 | 0.003 |
|  |  | Changes | 1.59 | 6.54 | 0.29 | 5.34 | 1.29 | 5.94 | 0.77 | 4.73 | 1.00 | 4.43 | 1.000 | 0.768 |  |
|  | Female | Baseline | 91.80 | 8.35 | 95.27 | 10.37 | 92.67 | 8.38 | 91.71 | 10.64 | 92.67 | 8.30 | 1.000 | 0.224 |  |
|  |  | 5^th^-year | 93.23 | 9.17 | 95.76 | 10.35 | 93.63 | 10.02 | 92.90 | 10.80 | 91.92 | 9.02 | 1.000 | 0.308 | 0.066 |
|  |  | Changes | 1.43 | 6.47 | 0.49 | 6.75 | 0.95 | 7.26 | 1.45 | 6.77 | -0.75 | 5.87 | 0.767 | 0.362 |  |
|  | Total | Baseline | 99.96 | 9.64 | 96.44 | 8.88 | 98.03 | 10.14 | 95.18 | 9.83 | 94.15 | 8.60 | <0.001 | <0.001 |  |
|  |  | 5^th^-year | 101.41^**^ | 9.35 | 97.13 | 9.90 | 98.78 | 9.78 | 95.89 | 10.97 | 94.50 | 9.50 | <0.001 | <0.001 | <0.001 |
|  |  | Changes | 1.02^**^ | 3.83 | 0.68 | 6.14 | 0.71 | 5.72 | 0.82 | 6.67 | 0.38 | 5.61 | 0.774 | 0.618 |  |
| WHtR (cm/m) | Male | Baseline | 61.45 | 4.90 | 60.79 | 4.67 | 59.68 | 4.57 | 60.77 | 5.10 | 59.88 | 4.78 | 0.872 | 0.286 |  |
|  |  | 5^th^-year | 62.32 | 5.57 | 60.97 | 4.76 | 60.44 | 4.68 | 61.20 | 5.36 | 60.47 | 5.30 | 0.640 | 0.320 | 0.003 |
|  |  | Changes | 0.98 | 3.97 | 0.18 | 3.17 | 0.76 | 3.48 | 0.47 | 2.83 | 0.60 | 2.70 | 1.000 | 0.755 |  |
|  | Female | Baseline | 59.68 | 5.74 | 61.43 | 7.51 | 60.23 | 5.53 | 60.24 | 7.09 | 60.16 | 6.04 | 1.000 | 0.665 |  |
|  |  | 5^th^-year | 60.57 | 5.82 | 61.70 | 7.08 | 60.81 | 6.43 | 60.98 | 7.42 | 59.69 | 6.62 | 1.000 | 0.598 | 0.075 |
|  |  | Changes | 0.89 | 4.30 | 0.28 | 4.34 | 0.59 | 4.72 | 0.98 | 4.48 | -0.47 | 3.81 | 0.909 | 0.376 |  |
|  | Total | Baseline | 60.84 | 5.09 | 60.07 | 5.80 | 60.95 | 5.83 | 60.37 | 5.98 | 59.92 | 5.74 | 1.000 | 0.572 |  |
|  |  | 5^th^-year | 61.80^**^ | 5.15 | 60.46 | 5.94 | 61.37 | 5.71 | 60.80 | 6.61 | 60.12 | 6.36 | 0.360 | 0.216 | 0.001 |
|  |  | Changes | 1.02^**^ | 3.83 | 0.38 | 3.89 | 0.45 | 3.53 | 0.51 | 4.35 | 0.25 | 3.60 | 1.000 | 0.618 |  |

Quintiles for male: Q1< 70.61; Q2: 70.62-88.94; Q3: 88.95-108.61; Q4: 108.62-137.11; Q5> 137.11; Quintiles for female: Q1< 91.67; Q2: 91.68-113.96; Q3: 113.97-138.28; Q4: 138.29-181.01; Q5>181.01; Quintiles for total: Q1<79.02; Q2: 79.03-99.50; Q3: 99.51-124.53; Q4: 124.54-160.06; Q5>160.06. TPE is expressed as mg GAE/ g creatinine.

TPE: total polyphenol excretion; GAE: gallic acid equivalent; BW: body weight; BMI: body mass index. WC: waist circumference; WHtR: waist-to-height ratio.

a. Data are given as means (SD); P < 0.05 indicates statistical significance. Values with asterisks are statistically different from baseline by paired-samples T-test (P < 0.05): *: P < 0.05; **: P < 0.01.

b. Data analyzed by Bonferroni post-hoc comparisons.

c. Data analyzed by ANOVA.

d. Data analyzed by paired-samples T-test.
