# Supplementary material for: Polyphenol Levels Are Inversely Correlated with Body Weight and Obesity in an Elderly Population after 5 Years of Follow Up (The Randomised PREDIMED Study)
Source: Nutrients. 2017 May 3;9(5):452. doi: 10.3390/nu9050452 (PMC5452182; doi:10.3390/nu9050452)
Supplement: Supplementary file 1 [file nutrients-09-00452-s001.zip › Supplementary Materials/Talbe S2. Multiple linear regression analyses with changes in anthropometric parameters and changes in quintiles of TPE after 5 years in the PREDIMED.docx]

**Table S2.** Multiple linear regression analyses with changes in anthropometric parameters and changes in quintiles of TPE after 5 years in the PREDIMED.

|  |  |  | β | SE | Beta | P | 95%CI |  |
| --- | --- | --- | --- | --- | --- | --- | --- | --- |
| Changes in BW | Male | Model 1 | -0.224 | 0.210 | -0.065 | 0.287 | -0.636 | 0.189 |
| (Kg) |  | Model 2 | -0.237 | 0.238 | -0.069 | 0.320 | -0.706 | 0.232 |
|  |  | Model 3 | -0.221 | 0.237 | -0.064 | 0.353 | -0.689 | 0.247 |
|  |  | Model 4 | -0.082 | 0.249 | -0.025 | 0.741 | -0.574 | 0.409 |
|  | Female | Model 1 | -0.502 | 0.193 | -0.150 | 0.010 | -0.882 | -0.121 |
|  |  | Model 2 | -0.775 | 0.223 | -0.231 | 0.001 | -1.214 | -0.335 |
|  |  | Model 3 | -0.669 | 0.225 | -0.199 | 0.003 | -1.112 | -0.226 |
|  |  | Model 4 | -0.723 | 0.239 | -0.215 | 0.003 | -1.193 | -0.253 |
|  | Total | Model 1 | -0.332 | 0.142 | -0.097 | 0.020 | -0.612 | -0.053 |
|  |  | Model 2 | -0.491 | 0.161 | -0.144 | 0.002 | -0.807 | -0.175 |
|  |  | Model 3 | -0.388 | 0.163 | -0.114 | 0.017 | -0.707 | -0.068 |
|  |  | Model 4 | -0.355 | 0.168 | -0.105 | 0.036 | -0.686 | -0.024 |
| Changes in BMI | Male | Model 1 | -0.083 | 0.074 | -0.068 | 0.262 | -0.229 | 0.063 |
| (Kg/m^2^) |  | Model 2 | -0.091 | 0.084 | -0.074 | 0.282 | -0.256 | 0.075 |
|  |  | Model 3 | -0.085 | 0.084 | -0.070 | 0.310 | -0.251 | 0.080 |
|  |  | Model 4 | -0.036 | 0.088 | -0.030 | 0.682 | -0.209 | 0.137 |
|  | Female | Model 1 | -0.196 | 0.083 | -0.136 | 0.019 | -0.360 | -0.032 |
|  |  | Model 2 | -0.306 | 0.096 | -0.212 | 0.002 | -0.496 | -0.116 |
|  |  | Model 3 | -0.261 | 0.097 | -0.181 | 0.008 | -0.452 | -0.069 |
|  |  | Model 4 | -0.283 | 0.103 | -0.196 | 0.006 | -0.487 | -0.080 |
|  | Total | Model 1 | -0.128 | 0.056 | -0.095 | 0.023 | -0.238 | -0.018 |
|  |  | Model 2 | -0.194 | 0.063 | -0.144 | 0.002 | -0.319 | -0.070 |
|  |  | Model 3 | -0.152 | 0.064 | -0.113 | 0.018 | -0.278 | -0.026 |
|  |  | Model 4 | -0.139 | 0.067 | -0.105 | 0.037 | -0.270 | -0.009 |
| Changes in WC | Male | Model 1 | -0.363 | 0.230 | -0.095 | 0.117 | -0.816 | 0.091 |
| (cm) |  | Model 2 | -0.253 | 0.264 | -0.066 | 0.339 | -0.772 | 0.267 |
|  |  | Model 3 | -0.237 | 0.264 | -0.062 | 0.370 | -0.758 | 0.283 |
|  |  | Model 4 | -0.169 | 0.267 | -0.047 | 0.528 | -0.695 | 0.357 |
|  | Female | Model 1 | -0.310 | 0.275 | -0.066 | 0.260 | -0.851 | 0.231 |
|  |  | Model 2 | -0.514 | 0.321 | -0.109 | 0.110 | -1.145 | 0.117 |
|  |  | Model 3 | -0.497 | 0.327 | -0.106 | 0.129 | -1.140 | 0.146 |
|  |  | Model 4 | -0.701 | 0.350 | -0.148 | 0.046 | -1.390 | -0.012 |
|  | Total | Model 1 | -0.310 | 0.180 | -0.072 | 0.086 | -0.664 | 0.044 |
|  |  | Model 2 | -0.367 | 0.205 | -0.085 | 0.074 | -0.769 | 0.035 |
|  |  | Model 3 | -0.320 | 0.209 | -0.075 | 0.126 | -0.731 | 0.090 |
|  |  | Model 4 | -0.363 | 0.216 | -0.086 | 0.094 | -0.788 | 0.062 |
| Changes in WHtR | Male | Model 1 | -0.222 | 0.138 | -0.098 | 0.108 | -0.494 | 0.049 |
| (cm/m) |  | Model 2 | -0.158 | 0.158 | -0.070 | 0.317 | -0.469 | 0.152 |
|  |  | Model 3 | -0.150 | 0.158 | -0.066 | 0.345 | -0.461 | 0.162 |
|  |  | Model 4 | -0.105 | 0.160 | -0.049 | 0.509 | -0.420 | 0.209 |
|  | Female | Model 1 | -0.195 | 0.179 | -0.064 | 0.277 | -0.549 | 0.158 |
|  |  | Model 2 | -0.317 | 0.210 | -0.103 | 0.132 | -0.729 | 0.096 |
|  |  | Model 3 | -0.306 | 0.214 | -0.100 | 0.154 | -0.726 | 0.115 |
|  |  | Model 4 | -0.446 | 0.229 | -0.144 | 0.053 | -0.897 | 0.005 |
|  | Total | Model 1 | -0.192 | 0.114 | -0.071 | 0.093 | -0.416 | 0.032 |
|  |  | Model 2 | -0.226 | 0.130 | -0.083 | 0.082 | -0.480 | 0.029 |
|  |  | Model 3 | -0.198 | 0.132 | -0.073 | 0.135 | -0.458 | 0.062 |
|  |  | Model 4 | -0.227 | 0.137 | -0.084 | 0.099 | -0.497 | 0.043 |

TPE: total polyphenol excretion (mg GAE/g creatinine); GAE: gallic acid equivalent; BW: body weight; BMI: body mass index. WC: waist circumference; WHtR: waist-to-height ratio.

Model 1. unadjusted; Model 2 was adjusted for baseline TPE and baseline BW; Model 3 adjusted as in Model 2 plus sex (only for total participants), age and intervention groups; Model 4 adjusted as in Model 3 plus smoking status (never, current, former), family history of CHD, physical activity, hypertension, diabetes, dyslipidemia, marital status (single, married, divorced, widowed), education level (primary school, high school, university), medication used (antihypertensive drugs, vitamins, insulin, oral hypoglycemic drugs, aspirin or other antiplatelet drug supplements taken in the last month) recruitment centers, 14-unit Mediterranean diet score and energy intake at baseline.
